# TF-chRDP: a method for simultaneously capturing transcription factor binding chromatin-associated RNA, DNA and protein

**DOI:** 10.3389/fcell.2025.1561540

**Published:** 2025-03-07

**Authors:** Duo Ning, Yuqing Deng, Tong Gao, Yang Yang, Gengzhan Chen, Simon Zhongyuan Tian, Meizhen Zheng

**Affiliations:** ^1^ Shenzhen Key Laboratory of Gene Regulation and Systems Biology, School of Life Sciences, Southern University of Science and Technology, Shenzhen, Guangdong, China; ^2^ Department of Systems Biology, School of Life Sciences, Southern University of Science and Technology, Shenzhen, Guangdong, China

**Keywords:** TF-chRDP, transcription factor, chromatin-associated RNA, DNA, protein

## Abstract

Transcription factors (TFs) play a crucial role in the regulation of gene expression and the structural organization of chromatin. They interact with proteins, RNA, and chromatin DNA to exert their functions. Therefore, an efficient and straightforward experimental approach that simultaneously captures the interactions of transcription factors with DNA, RNA, and proteins is essential for studying these regulatory proteins. In this study, we developed a novel method, TF-chRDP (Transcription Factor binding Chromatin-associated RNA, DNA, and Protein), which allows for the concurrent capture of these biomolecules in a single experiment. We enriched chromatin complexes using specific antibodies and divided the chromatin into three fractions: one for DNA library preparation to analyze the genomic binding sites of transcription factors, another for RNA library preparation to investigate the RNA associated with transcription factor binding, and the third for proteomic analysis to identify protein cofactors interacting with transcription factors. We applied this method to study the transcription factor p53 and its associated chromatin complexes. The results demonstrated high specificity in the enrichment of DNA, RNA and proteins. This method provides an efficient tool for simultaneously capturing chromatin-associated RNA, DNA and protein bound to specific TF, making it particularly useful for analyzing the role of protein-DNA-RNA complexes in transcriptional regulation.

## 1 Introduction

Transcription factors (TFs) are pivotal regulators of gene expression, orchestrating a wide array of biological processes by binding to specific DNA sequences and modulating the transcription of target genes (Lambert et al., 2018). Their activity is intricately linked to the structural organization of chromatin, which influences accessibility to DNA and the recruitment of the transcriptional machinery (Lu and Lionnet, 2021). The dynamic interactions between TFs, chromatin, and other biomolecules, such as RNA and proteins, are essential for the precise regulation of gene expression and cellular responses to various stimuli (Hudson and Ortlund, 2014; Oksuz et al., 2023).

Recent advances in molecular biology have underscored the importance of understanding the multifaceted roles of TFs in chromatin dynamics and gene regulation (Ramanathan et al., 2019). Traditional methods for studying TF interactions often focus on one type of biomolecule at a time, leading to a fragmented understanding of their functional networks. Conventional techniques, such as ChIP-seq (Chromatin immunoprecipitation followed by sequencing) (Park, 2009), CUT&RUN (Cleavage Under Targets and Release Using Nuclease) (Skene and Henikoff, 2017), and CUT&Tag (Cleavage Under Targets and Tagmentation) (Kaya-Okur et al., 2019), have been successfully used to identify TF-bound DNA loci. Methods like RIP-seq (RNA immunoprecipitation sequencing) (Zhao et al., 2010), CLIP-seq (Crosslinking immunoprecipitation coupled with high-throughput sequencing) (Yeo et al., 2009) and its derived methods (König et al., 2010; Van Nostrand et al., 2016; Zarnegar et al., 2016) help elucidate protein-bound RNA profiles. Additionally, ChIP-MS (Chromatin immunoprecipitation followed by mass spectrometry) (Wang et al., 2013) has been employed to investigate TF-associated chromatin proteins. Despite these advances, there remains a notable gap in methods that can simultaneously profile RNA, DNA and protein within the context of protein-DNA-RNA complexes, which is essential for understanding the intricate mechanisms of gene regulation.

To address this challenge, we developed a novel method, TF-chRDP (Transcription Factor binding Chromatin-associated RNA, DNA, and Protein), which facilitates the concurrent analysis of TF-bound RNA, DNA and proteins within a single experimental framework. This method enriches the chromatin DNA-RNA-protein complex using a specific antibody, enabling the dissection of TF interactions with their genomic targets, associated RNAs, and interacting protein cofactors within the complex. A key feature of the TF-chRDP method is the incorporation of Tn5 transposase, which efficiently constructs both RNA and DNA libraries by fragmenting DNA and adding DNA adapters in a single step. This dual functionality not only streamlines the library preparation process but also significantly enhances the efficiency of capturing low amounts of RNA and DNA obtained following antibody-based enrichment.

To demonstrate the utility of TF-chRDP, we applied this method to investigate protein-DNA-RNA complexes bounded to the transcription factor p53 in human colon cancer cells (HCT116). Under normal conditions, p53 protein is maintained at a low level due to degradation by the E3 ubiquitin ligase MDM2 (Hafner et al., 2019). To activate p53, we treated cells with the anti-cancer drug 5-fluorouracil (5-FU), which stabilizes and activates p53 (Longley et al., 2003). The results show that TF-chRDP provides high specificity in enriching DNA, RNA and proteins bound to a given TF, as demonstrated by comparisons with traditional ChIP-seq and RNA-seq results.

This protocol, which outlines detailed steps from cell culture and crosslinking to the preparation of DNA and RNA libraries, as well as protein identification, provides a highly efficient and straightforward approach for the simultaneous study of protein-DNA-RNA complex bound to specific TF. We believe this method will be a valuable tool for advancing our understanding of the functional roles of protein-DNA-RNA complexes in gene regulation.

## 2 Material and equipment

### 2.1 Reagents

- 0.5 M EDTA (Invitrogen, cat. No. AM9260G)
- 1 M Magnesium acetate (Sigma-Aldrich, cat. No. 63052-100 ML)
- 1 M Tris-HCl pH 7.0 (Invitrogen, cat. No. AM9851)
- 1 M Tris-HCl pH 8.0 (Invitrogen, cat. No. AM9856)
- 10% SDS (wt/vol) (Invitrogen, cat. No. AM9822)
- 5 M NaCl (Invitrogen, cat. No. AM9759)
- 5 M Potassium acetate (Sigma-Aldrich, cat. No. 95843-100 ML-F)
- 5× Second-Strand Reaction Buffer (Thermo Fisher, cat. No. 10812014)
- 5-Fluorouracil (Sigma-Aldrich, cat. No. F6627-1G)
- Acetonitrile (Sigma-Aldrich, cat. No. 1.00030)
- Ammonium Bicarbonate (NH_4_HCO_3_) (Sigma-Aldrich, cat. No. 09830)
- Ampure XP beads (Beckman, cat. No. A63881)
- Buffer EB (Qiagen, cat. No. 19086)
- Chloroform (Yonghua, cat. No. Y00042)
- cOmplete, Mini, EDTA-free Protease Inhibitor Cocktail (Roche, cat. No. 11836170001)
- Dimethyl sulfoxide (DMSO) (Sigma-Aldrich, cat. No. D2650-100 ML)
- Dithiothreitol (DTT) (Sigma-Aldrich, cat. No. D5545)
- DMEM, high glucose (Thermo Fisher, cat. No. 11965092)
- DNA Clean and Concentrator-5 (Zymo, cat. No. D4013)
- Dulbecco’s Phosphate Buffered Saline (DPBS) (Gibco, cat. No. 14190250)
- Dynabeads Protein G for Immunoprecipitation (Thermo Fisher, cat. No. 10004D)
- *E. coli* DNA Ligase (10 U/μL) (NEB, cat. No. M0205S)
- *E. coli* DNA Polymerase I (10 U/μL) (NEB, cat. No. M0209S)
- *E. coli* RNase H (2 U/μL) (Invitrogen, cat. No. 18080051)
- EDTA (0.5 M), pH 8.0, RNase-free (Thermo Fisher, cat. No. AM9260G)
- EGS [ethylene glycolbis (succinimidylsuccinate)] (Thermo Scientific, cat. No. 21565)
- Ethyl alcohol, pure (Sigma-Aldrich, cat. No. E7023-500 ML)
- Fetal Bovine Serum (Excell Bio, cat. No. FSP500)
- Formaldehyde solution (Sigma-Aldrich, cat. No. 47608-250 ML-F)
- Formic acid (Sigma-Aldrich, cat. No. 06473)
- HCT116 cell line (National Collection of Authenticated Cell Cultures, Shanghai Institute of Biochemistry and Cell Biology, Chinese Academy of Sciences, cat. No. TCHu 99)
- High Sensitivity Cartridge (N1) (BiOptic, cat. No. C105105)
- High-Fidelity 2× PCR Master Mix (NEB, cat. No. M0541)
- Iodoacetamide (IAM) (Sigma-Aldrich, cat. No. I6125)
- MIRNeasy micro kit (QIAGEN, cat. No. 217084)
- Mouse monoclonal anti-p53 (Santa Cruz Biotechnology, cat. No. sc-126)
- Nuclease-free water (not DEPC-Treated) (Invitrogen, cat. No. AM9937)
- Penicillin-Streptomycin (10,000 U/mL) (Thermo Fisher, cat. No. 15140122)
- Proteinase K Solution (Invitrogen, cat. No. AM2548)
- Qubit dsDNA HS Assay Kit (Invitrogen, cat. No. Q32851)
- RNase-Free DNase Set (QIAGEN, cat. No. 79254)
- RNaseZap™ RNase Decontamination Solution (Invitrogen, cat. No. AM9780)
- SuperScript™ III First-Strand Synthesis System (Invitrogen, cat. No. 18080051)
- T4 DNA Polymerase (3 U/μL) (NEB, cat. No. M0203S)
- TE Buffer pH 8.0 (Invitrogen, cat. No. AM9849)
- Triton X-100 (Acros Organics, cat. No. 327371000-100 mL)
- TRIzol™ Reagent (Thermo Fisher, cat. No. 15596026)
- Tn5 transposase (BGI, cat. No. LS-EZ-E−00009O)
- TruePrep™ Index Kit V2 for Illumina (Vazyme, cat. No. TD202)
- Trypsin-EDTA (0.25%), phenol red (Thermo Fisher, cat. No. 25200056)
- UltraPure Glycine (Invitrogen, cat. No. 50046-1 KG)

### 2.2 Equipment

- Analytical Balance (Sartorius, cat. No. BP211d)
- Automated Cell Counter (Counterstar, cat. No. IC1000)
- C1000 Touch Thermal Cycler (Bio-Rad, cat. No. 1851148)
- DynaMag-2 Magnet (Life Technologies, cat. No. 12321D)
- Easy-nLC 1,000 (Thermo Scientific, cat. No. LC120)
- Eppendorf 5424R Centrifuge (Eppendorf, cat. No. 5404F1621754)
- Eppendorf 5,425 Centrifuge (Eppendorf, cat. No. 5405000204)
- Eppendorf 5810R Centrifuge (Eppendorf, cat. No. 22628180)
- Eppendorf Thermomixer C (Eppendorf, cat. No. 5382000023)
- Intelli-mixer RM-2L (ELMI, cat. No. RM-2L)
- Low-Temperature High-Speed Centrifuge (Beckman Coulter, cat. No. Microfuge 22R)
- Magnetic Stirrer (CRYSTAL, cat. No. MS2-P1H)
- Micro Vac (TOMY, cat. No. MV-100)
- Microcentrifuge (HERMLE, cat. No. Z130M)
- Nikon Eclipse TS2 Microscope (Nikon, cat. No. Eclipse TS2)
- Orbitrap Exploris™ 240 Mass Spectrometer (Thermo Scientific, cat. No. BRE725535)
- Qsep Bio-Fragment Analyzer (BiOptic, cat. No. Qsep100)
- Qubit 4.0 Fluorometer (Invitrogen, cat. No. Q33226)
- Refrigerator (Haier, cat. No. HYC-940)
- Refrigerator (Haier, cat. No. DW-25L262)
- Heracell VIOS 160i CO_2_ Incubator (Thermo Scientific, cat. No. 51033549)
- Ultra-Low Temperature Freezers (Thermo Scientific, cat. No. 995)
- Ultrasonic Processor (Sonics, cat. No. VCX130)
- Vacuum Centrifugal Concentrator (Eppendorf, cat. No. Concentrator plus)
- Vortex 6 (Kylin-Bell)

### 2.3 Reagents set-up.

#### 2.3.1 375 mM 5-FU solution stock

Add 48.8 mg of 5-FU powder to a 1.5-mL tube, and then add 1 mL of DMSO; mix the solution well. Store the solution at 4°C, away from light. The solution can be stored at 4°C for up to 1 month.

#### 2.3.2 1% formaldehyde (FA)/DPBS

Add 48.62 mL of DPBS to a 50-mL tube first, and then add 1.38 mL of 36% formaldehyde; mix the solution well. The solution can be pre-warmed at 37°C.

▲ CRITICAL: Manipulate formaldehyde in a chemical hood. Prepare the solution freshly.

#### 2.3.3 2.5 M glycine solution

Add 9.375 g of Glycine powder to a 50-mL tube, and then add DPBS up to 50 mL; mix the solution well. The solution can be stored at room temperature (RT) for up to 1 year.

#### 2.3.4 2 mM EGS solution

Add 45.63 mg of EGS powder to a 1.5-mL tube, and then add 200 µL of DMSO. Mix the solution well away from light. After all the powder has dissolved in DMSO, transfer the EGS solution to a 50-mL tube containing 49.8 mL of DPBS, and mix the solution well. The buffer can be pre-warmed in a 37°C incubator.

▲ CRITICAL: Manipulate DMSO in a chemical fume hood. Prepare the solution freshly.

#### 2.3.5 20% triton X-100

Add 40 mL of nuclease-free water to a 50-mL tube first, and then slowly add 10 mL of Triton X-100; mix the solution well by rotation overnight. The buffer can be stored at RT for up to 6 months.

▲ CRITICAL: Triton X-100 is highly viscous, aspirate and dispense it slowly using pipette tips. Pipette up and down several times to ensure all Triton X-100 adhering to the tip walls is recovered. Protect the buffer from light.

#### 2.3.6 0.1% SDS FA buffer

Add 45.4 mL of nuclease-free water to a 50-mL tube first, and then add sequentially 1.25 mL of 1 M Tris-HCl pH 7.0, 1.25 mL of 1 M Tris-HCl pH 8.0, 1.5 mL of 5 M NaCl, 100 µL of 0.5 M EDTA and 500 µL of 10% SDS (wt/vol); mix the solution well. The buffer can be stored at RT for up to 6 months.

#### 2.3.7 Low salt buffer

Add 42.9 mL of nuclease-free water to a 50-mL tube first, and then add sequentially 1.25 mL of 1 M Tris-HCl pH 7.0, 1.25 mL of 1 M Tris-HCl pH 8.0, 1.5 mL of 5 M NaCl, 100 µL of 0.5 M EDTA, 2.5 mL of 20% Triton X-100 and 500 µL of 10% SDS (wt/vol); mix the solution well. The buffer can be stored at 4°C for up to 6 months.

#### 2.3.8 High salt buffer

Add 40.9 mL of nuclease-free water to a 50-mL tube first, and then add sequentially 1.25 mL of 1 M Tris-HCl pH 7.0, 1.25 mL of 1 M Tris-HCl pH 8.0, 3.5 mL of 5 M NaCl, 100 µL of 0.5 M EDTA, 2.5 mL of 20% Triton X-100 and 500 µL of 10% SDS (wt/vol); mix the solution well. The buffer can be stored at 4°C for up to 6 months.

#### 2.3.9 0.1% triton X-100/DPBS

Add 49.75 mL of DPBS to a 50-mL tube first, and then add 250 µL of 20% Triton X-100; mix the solution well. The buffer can be stored at 4°C for up to 6 months.

#### 2.3.10 RNA proteinase K buffer

Add 9.18 mL of nuclease-free water to a 15-mL tube first, and then add sequentially 100 µL of 1 M Tris-HCl pH 7.0, 200 µL of 5 M NaCl, 20 µL of 0.5 M EDTA and 500 µL of 10% SDS (wt/vol); mix the solution well. The buffer can be stored at RT for up to 6 months.

#### 2.3.11 1 × protease inhibitor (PI) solution

Add 1 tablet of Protease Inhibitor Cocktail to a 1.5-mL tube, and then add 500 µL of nuclease-free water; mix the solution well. The buffer can be stored at −20°C for up to 6 months.

#### 2.3.12 DNase I incubation mix

Add 70 µL of Buffer RDD to a 1.5-mL tube first, and then add 10 µL of DNase I stock solution; mix by gently inverting the tube. Place the tube on ice before use.

▲ CRITICAL: Prepare the mix freshly.

#### 2.3.13 80% ethanol solution

Add 2 mL of nuclease-free water to a 15-mL tube first, and then add 8 mL of pure ethanol; mix the solution well.

▲ CRITICAL: Prepare the solution freshly.

#### 2.3.14 4 × THS TD buffer

Add 676 µL of nuclease-free water to a 15-mL tube first, and then add sequentially 660 µL of 1 M Tris-HCl pH 8.0, 264 µL of 5 M Potassium acetate, 200 µL of 1 M Magnesium acetate and 3.2 mL of DMF; mix the solution well. The buffer can be stored at −20°C for up to 1 year.

▲ CRITICAL: Manipulate DMF in a chemical hood.

## 3 Methods

### 3.1 Cell culture (TIMING ∼1 week)

HCT116 human colorectal cancer cell line (male) was purchased from the National Collection of Authenticated Cell Cultures, Shanghai Institute of Biochemistry and Cell Biology, Chinese Academy of Sciences. The cells were cultured in DMEM medium supplemented with 10% fetal bovine serum (FBS), 100 units/mL of penicillin, and 100 μg/mL of streptomycin at 37°C with 5% CO_2_.

### 3.2 5-FU treatment (TIMING ∼10 h)

Upon reaching a cell density of approximately 70% in the 15 cm dish, the cell culture medium was replaced with fresh medium containing 375 μM 5-FU. The cells were then incubated for 9 h. As a control, cells were treated with fresh culture medium supplemented with an equivalent amount of DMSO and incubated for 9 h at 37°C with 5% CO_2_.

### 3.3 Cell crosslinking (TIMING ∼3 h)

#### 3.3.1 1% formaldehyde (FA) crosslinking

Remove the culture medium from the 15 cm dishes and wash the cells twice with 20 mL of Dulbecco’s Phosphate-Buffered Saline (DPBS), shaking the plate on the rotator for 5 min during each wash at RT, 50 rpm. Remove the DPBS and add 20 mL of 1% formaldehyde (FA) solution to the dish. Shake the dish on the rotator for 20 min at RT, 50 rpm. Then, directly add 1.74 mL of 2.5 M glycine to quench the reaction (to a final concentration of 0.2 M) and rotate at RT for 10 min, 50 rpm. Discard the supernatant and wash the dish twice with 20 mL of DPBS, shaking on the rotator for 5 min during each wash at RT, 50 rpm.

#### 3.3.2 EGS crosslinking

Remove the DPBS and add 20 mL of 2 mM EGS to the dish. Shake the dish on the rotator for 45 min at RT, 50 rpm. Then, directly add 1.74 mL of 2.5 M glycine to quench the reaction (to a final concentration of 0.2 M) and rotate at RT for 10 min, 50 rpm. Discard the supernatant and wash the dish twice with 20 mL of DPBS, shaking on the rotator for 5 min during each wash at RT, 50 rpm.

#### 3.3.3 Collect the dual-crosslinked cells

After the second DPBS wash, place the dish on ice. Remove the DPBS, and rinse the dish with cold DPBS containing PI solution. Scrape the cells on ice, collect approximately 20 million cells from each 15 cm dish, and pool the cells from all dishes with the same treatment into a 15-mL tube. Spin down the tube at 2,000 rpm for 5 min at 4°C. Discard the supernatant and resuspend the cells in DPBS. Count the cell number and aliquot them into 15-mL tubes, with each tube containing approximately 30 million cells. Spin down the cells at 5,000 rpm for 5 min at 4°C, discard the supernatant.

▲ **PAUSE POINT:** Store the cell pellet at −80°C for future use.

### 3.4 Protein G beads preparation (TIMING ∼9 h)

Prepare 150 μL of Protein G Beads per antibody for immunoprecipitation (IP). Wash the beads three times with 1 mL 0.1% Triton X-100/DPBS. For each sample, add 10 µg of anti-p53 antibody to the washed Protein G beads in 500 µL of 0.1% Triton X-100/DPBS. Rotate the tube at 4°C (F1, 12 rpm) over 8 h for binding of antibody with the Protein G beads.

For each sample, take 50 μL of Protein G beads and wash them three times with 1 mL of 0.1% Triton X-100/DPBS buffer. After washing, store the beads in 500 μL of 0.1% Triton X-100/DPBS at 4°C until ready for chromatin preclearing.

### 3.5 Cell lysis and nuclear permeabilization (TIMING ∼2 h)

#### 3.5.1 Cell lysis

Thaw the 1% FA and 2 mM EGS crosslinked cells on ice for 20 min, using 30 million cells per sample. Add 10 mL of 0.1% SDS FA (supplemented with PI) to the cell pellet, mix well by hand, and rotate the tube at RT (F1, 11 rpm) for 15 min.

#### 3.5.2 Nuclear permeabilization

Then, directly add 1 mL of 10% SDS and rotate the sample at 37°C for 20 min. Check cell state under a microscopy at 10 min and 15 min. Spin down at 2,000 rpm for 10 min at 4°C and discard the supernatant. Wash the nuclei with 10 mL of 0.1% SDS FA buffer by incubating on the Intelli-Mixer (F1, 20 rpm) for 5 min at RT.

### 3.6 Sonication (TIMING ∼1 h)

Spin down the sample at 4°C, 12,000 rpm for 15 min to remove supernatant. Suspend the cell pellet in 3 mL of 0.1% SDS cell lysis buffer (supplemented with PI). Transfer 1 mL of the sample to a new 14-mL Falcon round-bottom tube and place on ice. Prepare a total of three tubes for each sample.

Clean the sonication tip sequentially with sterilized water, 75% ethanol, and sterilized water. Immerse the sonication tip in nuclease-free water and sonicate for 1 min to further clean the tip. Immerse the sample tubes in a water-ice mixture and start sonication. The sonication parameters are: amplitude 38%, total time 5 min, with on/off cycles of 30 s each.

After sonication, transfer the samples back to their original 15-mL tubes and centrifuge at 4°C, 2,000 rpm for 15 min. Collect the supernatant (chromatin) into new 1.5-mL tubes (1 mL chromatin per tube).

### 3.7 Preclear chromatin (TIMING ∼1 h)

Aliquot the pre-washed Protein G beads and mix them with the collected chromatin suspension. Incubate the mixture at 4°C for at least 1 h with rotation (F1, 12 rpm) to remove any non-specific binding materials.

### 3.8 IP overnight (TIMING ∼12 h)

Take two 10 µL aliquots of the sample after preclearing for quality control (QC): one for DNA QC and one for input RNA QC, and store them at −20°C. Wash the remaining antibody-bound Protein G beads twice with 1 mL of 0.1% Triton X-100/DPBS buffer using a magnetic stand. Discard the wash buffer. Add the pre-cleared chromatin suspension to the washed antibody-bound Protein G beads for the IP process. Incubate the mixture with rotation at 4°C (F1, 12 rpm) overnight.

### 3.9 Wash the chromatin complex after IP (TIMING ∼2 h)

After overnight IP, remove and discard the supernatant using a magnetic stand. Wash the antibody-captured chromatin complex on the Protein G beads with Low salt buffer three times, High salt buffer twice, TE buffer twice. For each wash, add 1 mL of wash buffer to resuspend beads, place the tube on a rotating rack (F1, 12 rpm) at 4°C for 5 min, perform a brief centrifugation, and place the tube back on the magnetic rack, then remove the supernatant. In the final wash step, aliquot the beads into 3 separate tubes for the preparation of the DNA library, RNA library, and protein identification.

### 3.10 TF-chRDP protein identification with nano liquid chromatography-tandem mass spectrometry (LC-MS/MS) (TIMING ∼3 days)

#### 3.10.1 Trypsin digestion

Place the tube on a magnetic rack and remove the TE buffer. Wash the beads twice with 200 μL of DPBS, discarding the supernatant each time. Resuspend the beads in 100 μL of 50 mM NH_4_HCO_3_, add DTT solution to a final concentration of 10 mM and incubate at 56°C for 1 h. Add IAM solution to a final concentration of 50 mM and incubate at RT in dark for 1 h. Add Trypsin to the protein solution at a mass ratio of 1:100 and digest at 37°C for 4 h. Add Trypsin again at a mass ratio of 1:100 and continue the digestion at 37°C overnight (16 h).

#### 3.10.2 Desalting and peptide preparation

Remove the salt from the sample using a home-made C18 tip, and dry the solvent in a vacuum concentrator at 45°C. Dissolve the peptides in 0.1% formic acid, fully vortex the solution and centrifuge at 13,200 rpm for 10 min at 4°C. Transfer the supernatant to the sample tube and proceed with mass spectrometry (MS) analysis.

#### 3.10.3 LC-MS/MS analysis

The protein samples were analyzed using a Nanoflow UPLC system, with 4 μL of sample loaded for each analysis. Mobile phase A consisted of 0.1% formic acid in water, while mobile phase B was composed of 20% 0.1% formic acid in water and 80% acetonitrile. The total flow rate during the analysis was set to 600 nL/min. The LC linear gradient was as follows: 0 min–6% phase B, 5 min–9% phase B, 20 min–14% phase B, 50 min–30% phase B, 58 min–40% phase B, and 60 min–95% phase B.

The mass spectrometry was performed using the Orbitrap Exploris™ 240 Mass Spectrometer. The spray voltage was set at 2.2 kV, and the capillary temperature was maintained at 320°C. The MS resolution was 60,000 at 400 m/z, with a precursor m/z range of 350–1,500. For MS/MS analysis, HCD (High Collision Dissociation) activation was applied with a normalized collision energy of 30 and an activation time of 60 m. Data-dependent MS/MS was performed on the top 20 most intense peptide ions from the preview scan in the Orbitrap.

### 3.11 Decrosslinking (TIMING ∼12 h)

#### 3.11.1 Decrosslinking of TF-chRDP DNA and QC sample

For the TF-chRDP DNA, after removing the TE buffer, add 40 μL of TE buffer, 50 μL of ChIP elution buffer and 10 μL of Proteinase K to the beads. Incubate at 65°C overnight with shaking at 900 rpm.

For the DNA QC sample, add 30 μL of TE buffer, 50 μL of ChIP elution buffer and 10 μL of Proteinase K to 10 μL of QC sample. Incubate at 65°C overnight with shaking at 900 rpm.

▲ **PAUSE POINT:** Store the decrosslinked sample at −20°C, or proceed with DNA purification.

#### 3.11.2 Decrosslinking of TF-chRDP RNA and QC sample

For the TF-chRDP RNA, after removing the TE buffer, add 95 μL of RNA proteinase K buffer and 5 μL of Proteinase K to the beads. Incubate at 50°C for 45 min with shaking at 900 rpm.

For the input RNA QC sample, add 85 μL of RNA proteinase K buffer and 5 μL of Proteinase K to 10 μL of QC sample. Incubate at 50°C for 45 min with shaking at 900 rpm.

Briefly spin down all tubes and boil the samples for 10 min on a heat block at 95°C. Chill the samples on ice for 5 min, then add 500 μL of TRIzol and vortex vigorously for 10 s. Incubate at RT for 10 min.

▲ **PAUSE POINT:** Store the decrosslinked sample at −80°C, or proceed with RNA purification.

### 3.12 DNA purification for TF-chRDP DNA and QC samples (TIMING ∼1 h)

Purify the DNA QC sample and TF-chRDP DNA using Zymo Genomic DNA Clean and Concentrator Kit-10 according to the manufacturer’s instructions. Briefly, place the TF-chRDP DNA sample on a magnetic rack for 2 min and transfer the supernatant to a new tube. Add five volumes of DNA Binding Buffer to both the TF-chRDP DNA sample and QC samples. Mix briefly by vortexing, and then spin down briefly. Transfer the mixture to Zymo-Spin Column in a Collection Tube and spin down at 13,000 rpm for 30 s at RT. Run the flow through into column again, at 13,000 rpm, RT for 30 s. Discard the flow-through. Add 200 μL of DNA Wash Buffer to the Column, spin down at 13,000 rpm at RT for 30 s. Add another 200 μL of DNA Wash Buffer to the Column and spin down at 13,000 rpm at RT for 30 s. Discard the flow-through. Spin down the Column at 13,000 rpm at RT for 1 min to remove any residual Wash Buffer. Transfer the Column to a new 1.5-mL Low-binding collection tube. Add 15 μL of Buffer EB directly to the top of the Column filter; incubate at RT for 2 min, then spin down at 13,000 rpm for 1 min at RT. Collect the 14 μL of eluted DNA in the 1.5-mL low-binding tube.

Quantify the DNA concentration using Qubit 4.0, and analyze the DNA fragment distribution of QC sample using Qsep Bio-Fragment Analyzer.

▲ **PAUSE POINT:** Store the purified DNA at −20°C, or proceed with DNA library preparation.

### 3.13 TF-chRDP DNA library preparation and sequencing (TIMING ∼10 days)

#### 3.13.1 Tn5 testing

Set up the Tn5 testing reaction in a 200-μL tube on ice: add 6.67 μL of nuclease-free water, 8 μL of QC DNA (4 ng), 5 μL of 4× THS TD buffer and 0.33 μL of Tn5 Transposase. Mix well with a pipette and incubate at 55°C for 10 min, then cool down at 4°C.

Directly add 3 μL of 0.2% SDS solution to the reaction, mix well, and incubate at 37°C for 5 min, RT for 5 min, and then place on ice for more than 5 min. Set up the PCR reaction on ice: add 1 μL of Index one (i7), 1 μL of Index two (i5) and 25 μL of High-Fidelity 2× PCR Master Mix. Pipette to mix well, and incubate in the PCR instrument. Start the following PCR programs: Heat the lid at 105°C, incubate at 72°C for 3 min, then incubate at 98°C for 30 s. Perform eight cycles of the following three steps: incubate at 98°C for 15 s; incubate at 60°C for 30 s; incubate at 72°C for 3 min. Finally, incubate at 72°C for 5 min and hold at 4°C. After PCR, purify the product with 1× volume Ampure XP beads.

Quantify the DNA concentration using Qubit 4.0, and analyze the DNA fragment distribution using Qsep Bio-Fragment Analyzer. If the PCR product size is between 250 and 600 bp and the DNA amount is more than 30 ng, the Tn5 testing condition is suitable for the TF-chRDP DNA sample.

#### 3.13.2 Tagmentation and PCR

Set up the Tn5 tagmentation and PCR reactions according to the Tn5 testing conditions for the TF-chRDP DNA sample.

#### 3.13.3 Double size selection

Take the PCR products and Ampure XP beads to RT more than 20 min for balance. Add Buffer EB to the PCR product to reach a final volume of 100 μL. Mix the Ampure XP beads well and add 61 μL (0.61× volume) Ampure XP beads to the sample. Incubate on an Intelli-mixer with rotation (UU, 50 rpm) for 10 min. Place the tube on the magnetic rack for 5 min and collect the supernatant into a new tube. Next, directly add 19 μL ((0.8–0.61) × volume) of Ampure XP beads to the collected supernatant. Incubate on the Intelli-mixer with rotation (UU, 50 rpm) for 10 min. Place the tube on the magnetic rack for 5 min and discard the supernatants. Add 800 μL of 80% ethanol and spin the tubes 180° (5 times) to wash the beads. Remove the supernatant and repeat the wash with the 80% ethanol again. Remove the supernatant completely, using a 10 μL pipette to remove any remaining liquid. Vacuum centrifuge the sample for about 30 s, checking at intervals until a crevice forms on the beads. Add 8 μL of Buffer EB to elute the DNA. Mix well and rotate on the Intelli-mixer (uu, 70 rpm) for 10 min. Place the tube on the magnetic rack and carefully transfer 6 μL of the supernatant to a new 1.5-mL tube. Quantify the DNA concentration using Qubit 4.0.

▲ **PAUSE POINT:** Store the TF-chRDP DNA library at −20°C.

#### 3.13.4 Sequencing

The TF-chRDP DNA libraries were sequenced with 150 bp paired-end reads on the Illumina NovaSeq 6,000 platform.

### 3.14 RNA purification for TF-chRDP RNA and input RNA QC samples (TIMING ∼2 h)

#### 3.14.1 RNA extraction and precipitation

Add 100 μL of chloroform to TRIzol treated samples. Vortex vigorously for 10 s. Spin down at 16,100 rcf for 15 min at 4°C. Carefully transfer 400 μL of the aqueous supernatant, avoiding organic (pink) and interface, into a fresh tube. Add 600 μL of 100% ethanol and mix well.

#### 3.14.2 RNA purification with MIRNeasy micro kit

Purify RNA using a MIRNeasy micro kit, follow the guide in the MIRNeasy Micro Handbook. Pipette up to 700 µL of the sample, into an RNeasy MinElute spin column in a 2-mL collection tube. Close the lid gently and centrifuge at 10,000 rpm for 15 s at RT. Discard the flow-through. Repeat last step using the remainder of the sample. Discard the flow-through. Pipette 350 µL of Buffer RWT into the RNeasy MinElute spin column and centrifuge for 15 s at 10,000 rpm to wash. Discard the flow-through.

#### 3.14.3 On-column DNase digestion

Pipette 80 µL of DNase I incubation mix directly onto the RNeasy MinElute spin column membrane and incubate on the benchtop at 20–30°C for 15 min.

#### 3.14.4 Wash the column after DNase digestion

Add 500 µL of Buffer RWT into the RNeasy MinElute spin column and centrifuge for 15 s at 10,000 rpm. Keep the flow-through. Reapply the flow-through to the RNeasy MinElute spin column and centrifuge for 15 s at 10,000 rpm. Discard the flow-through. Add 500 µL Buffer RPE onto the RNeasy MinElute spin column. Close the lid gently and centrifuge for 15 s at 10,000 rpm to wash the column. Discard the flow-through. Pipette 500 µL of 80% ethanol onto the RNeasy MinElute spin column. Close the lid gently and centrifuge for 2 min at 10,000 rpm to wash the spin column membrane. Discard the collection tube with the flow-through.

#### 3.14.5 RNA elution and DNase inactivation

Place the RNeasy MinElute spin column into a new 2-mL collection tube. Open the lid of the spin column and centrifuge at full speed for 5 min to dry the membrane. Discard the collection tube with the flow-through. Place the RNeasy MinElute spin column in a new 1.5-mL collection tube. Add 14 µL of RNase-free water directly to the center of the spin column membrane. Close the lid gently and centrifuge for 1 min at full speed to elute the RNA. After the reaction is complete, heat the sample for 15 min at 65°C to completely inactivate any remaining DNase.

### 3.15 First-strand cDNA synthesis (TIMING ∼1.5 h)

Transfer 13 µL of RNA to a 200-μL tube, add 1 μL of Random hexamers (50 ng/μL) and 1 μL of 10 mM dNTP mix, pipette the mixture well and incubate the tube at 65°C for 5 min, then place on ice for at least 1 min. Prepare the cDNA Synthesis Mix by sequentially adding 4 μL of nuclease-free water, 3 μL of 10× RT buffer, 4 μL of 25 mM MgCl_2_, 2 μL of 0.1 M DTT, 1 μL of RNaseOUT (40 U/μL) and 1 μL of SuperScript III RT (200 U/μL) to the sample, mix gently, and collect by brief centrifugation. Incubate as follows: 10 min at 25°C, followed by 50 min at 50°C. After the reaction, chill on ice and collect the reactions by brief centrifugation.

### 3.16 Second-strand cDNA synthesis (TIMING ∼2.5 h)

Place the tube containing 30 μL of the first-strand reaction sample on ice. Sequentially add the following reagents to the first-strand reaction tube: 81 μL of nuclease-free water, 30 μL of 5× Second-Strand Reaction Buffer, 3 μL of 10 mM dNTP mix, 1 μL of *E. coli* DNA Ligase (10 U/μL), 4 μL of *E. coli* DNA Polymerase I (10 U/μL) and 1 μL of *E. coli* RNase H (2 U/μL). Mix well and incubate for 2 h at 16°C, ensuring the temperature does not rise above 16°C. Add 3.3 μL of T4 DNA Polymerase (3 U/μL) to each reaction and continue incubation on a Thermomixer (1,100 rpm) at 12°C for 15 min. Place the tube on ice and add 10 μL of 0.5 M EDTA.

### 3.17 cDNA purification (TIMING ∼1 h)

Take the cDNA sample and Ampure XP beads to RT for at least 20 min for balance. Mix the Ampure XP beads well and add 294 μL (1.8× volume) Ampure XP beads to the cDNA sample. Incubate on Intelli-mixer with rotation (UU, 50 rpm) for 10 min. Place the tube on the magnetic rack for 5 min and then remove the supernatant. Add 800 μL of 80% ethanol and spin the tubes 180° (5 times) to wash the beads. Remove the supernatant and repeat the wash with 80% ethanol. Remove the supernatant completely with a 10 μL pipette. Perform vacuum centrifugation for approximately 3 min, checking at intervals until a crevice forms on the beads. Add 14 μL of Buffer EB to elute the DNA. Mix well and rotate on the Intelli-mixer (uu, 70 rpm) for 10 min. Place the tube on the magnetic rack and carefully collect 12 μL of the supernatant into a new tube.

Quantify the DNA concentration by Qubit 4.0, and analyze the DNA fragment distribution by Qsep Bio-Fragment Analyzer.

▲ **PAUSE POINT:** Store the cDNA sample at −20°C.

### 3.18 TF-chRDP RNA library preparation and sequencing (TIMING ∼10 days)

After obtaining the purified double-stranded cDNA, Tn5 transposition was used for RNA library preparation. Perform the Tn5 testing using the input cDNA samples following the similar steps outlined in section 3.13.1. If the testing result is as expected, precede to the Tn5 tagmentation and double size selection steps as described in section 3.13 for TF-chRDP RNA samples. The TF-chRDP RNA libraries were sequenced with 150 bp paired-end reads on the Illumina NovaSeq 6,000 platform.

### 3.19 Data processing and visualization for TF-chRDP DNA and RNA (TIMING ∼3 days)

The TF-chRDP DNA library and downloaded published ChIP-seq (Sánchez et al., 2014) FASTQ files were analyzed using the ChIA-PIPE pipeline (Lee et al., 2020), with data aligned to the hg38 reference genome. Protein binding peaks were called using MACS2 (v2.2.6), and peak overlap analysis was performed using bedtools intersect (v2.29.2) (Quinlan and Hall, 2010). Read counts at peak locations were obtained using bedtools coverage (v2.29.2).

The TF-chRDP RNA library FASTQ files were first trimmed to remove adapters and then aligned to the hg38 reference genome using STAR (v2.7.10b) (Dobin et al., 2013). Gene and transcript expression quantification was performed using RSEM (v1.2.28) (Li and Dewey, 2011), followed by differential expression analysis using edgeR (Robinson et al., 2010). Significantly enriched RNAs were filtered with logFC >6.

DNA binding density and RNA profiling were visualized using the BASIC Browser (Lee et al., 2020).

### 3.20 Data analysis for TF-chRDP protein identification (TIMING ∼3 days)

The raw MS files were analyzed and searched against the target protein database corresponding to the species of the samples using MaxQuant (v1.6.2.10) (Tyanova et al., 2016). The parameters for the analysis were set as follows: carbamidomethylation (a fixed modification of cysteine), oxidation (a variable modification of methionine), and acetylation (a variable modification at the protein’s N-terminal). Trypsin was specified as the enzyme, with a maximum of two missed cleavages allowed. The precursor ion mass tolerance was set to 20 ppm, and the MS/MS tolerance was also set to 20 ppm.

## 4 Results

### 4.1 Overview of the TF-chRDP method

**FIGURE 1:**
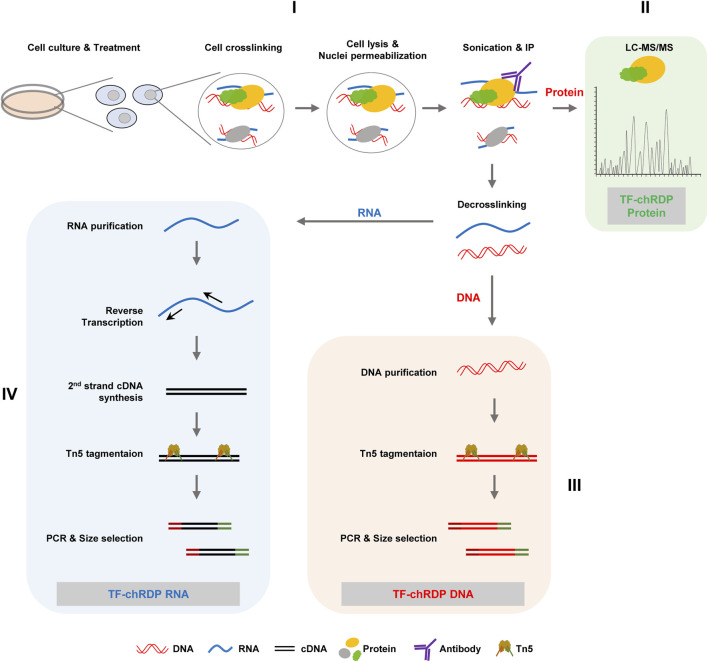
The overall workflow of TF-chRDP is illustrated. **(I)** Following treatment, cells were subjected to 1% FA and 2 mM EGS dual-crosslinking to preserve the protein-DNA-RNA complexes in the nuclei. After cell lysis and nuclear permeabilization, the complexes were sonicated to release them into solution and then enriched using a specific antibody. **(II)** The antibody-enriched complexes were aliquoted into three parts: one for protein identification by LC-MS/MS, and the other two for DNA and RNA library preparation after decrosslinking. **(III)** For TF-chRDP DNA library preparation, DNA was purified and tagmented using Tn5 transposase. After PCR amplification and double-size selection, the TF-chRDP DNA libraries were prepared for sequencing. **(IV)** For TF-chRDP RNA library preparation, RNA was purified and reverse-transcribed into cDNA. The double-stranded cDNA was then used for library construction, following a similar approach to DNA library preparation.

The TF-chRDP (Transcription Factor binding Chromatin-associated RNA, DNA, and Protein) method is designed to simultaneously capture and analyze the interactions of transcription factors with chromatin-associated RNA, DNA, and proteins in a single experimental framework (Figure 1). The process begins with cell culture and treatment to induce the expression of the transcription factor of interest, followed by crosslinking to stabilize interactions between TFs and their associated biomolecules. After cell lysis and chromatin preparation, sonication fragments the chromatin, and immunoprecipitation using specific antibodies enriches the TF-chromatin complexes. The enriched complexes are aliquoted into three parts: one for protein identification *via* LC-MS/MS, and the other two for DNA and RNA analysis following decrosslinking.

For the TF-chRD DNA library preparation, the purified DNA is tagmented with Tn5 transposase for DNA fragmentation and the addition of sequencing adapters. The Tn5-tagmented samples are directly used for PCR amplification and subsequent double size selection. For the TF-chRD RNA library preparation, RNA is purified and reverse transcribed using random hexamers. After second-strand cDNA synthesis, the double-stranded cDNA is used for library construction, utilizing Tn5 transposase in a similar method to DNA library construction. The TF-chRD DNA and RNA libraries are sequenced with 150 bp paired-end reads on the NovaSeq 6,000 sequencer.

### 4.2 Assessing the specificity of TF binding to genomic DNA using the TF-chRDP method

**FIGURE 2:**
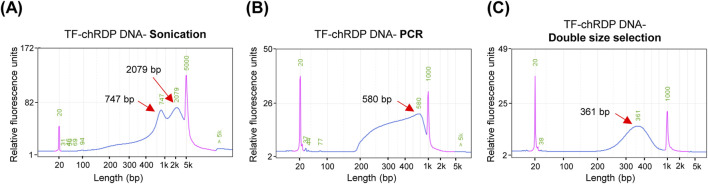
TF-chRDP DNA library preparation. **(A)** DNA fragment size distribution after sonication. **(B)** DNA fragment size distribution following PCR amplification of the TF-chRDP DNA. **(C)** DNA fragment size distribution after double size selection of the TF-chRDP DNA library. Red arrows indicate the peak positions, and the purple peak represents the marker.

The TF-chRDP method for DNA library preparation involves several key steps, as illustrated in Figure 2. The fragment size of sonicated samples showed that most chromatin DNA was sheared to a range of 700–2,000 bp (Figure 2A). The purified TF-bound DNA was then used for Tn5 tagmentation and PCR amplification. To achieve a suitable PCR product size distribution (300–600 bp), which is optimal for sequencing and improves size selection efficiency, we tested the appropriate amount of Tn5 transposase for the TF-chRDP DNA (Figure 2B).

In this study, we simplified the procedure by quenching the Tn5 reaction and directly proceeding to PCR without an additional DNA purification step. This was achieved by adding a low concentration of SDS to release the Tn5 transposase, which did not interfere with subsequent PCR steps, thereby eliminating the need for extra DNA purification. After double size selection, the library size was predominantly between 250 and 600 bp (Figure 2C).

**FIGURE 3:**
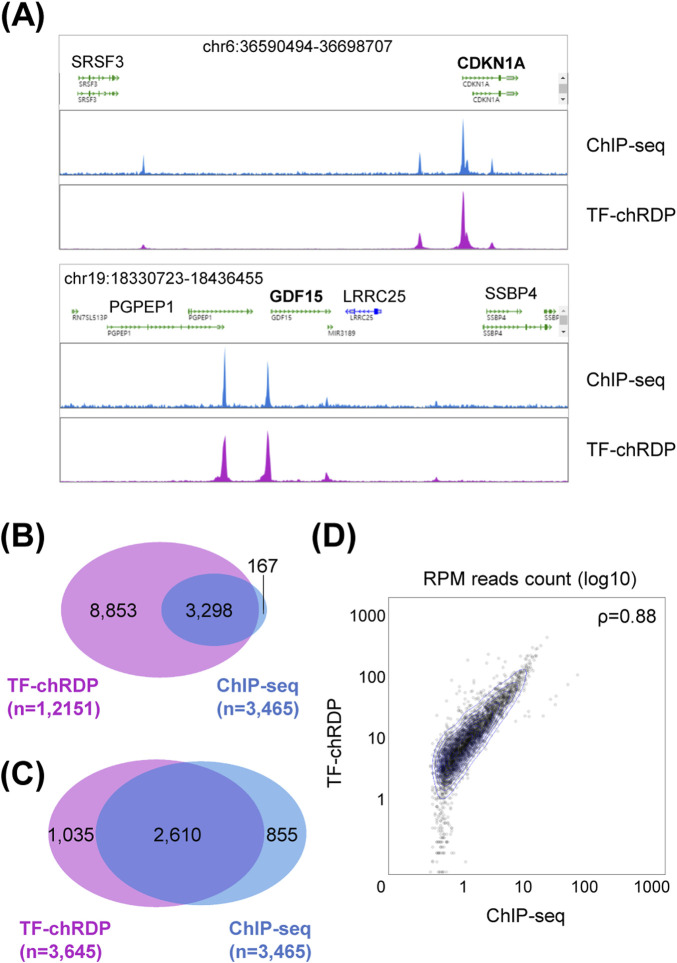
TF-chRDP DNA results show high specificity and consistent results with ChIP-seq. **(A)** Screenshots of Basic Browser showing the comparison of TF-chRDP and ChIP-seq results at the p53 classical target gene *CDKN1A* (top) and *GDF15* (bottom) regions. **(B)** Venn diagram showing the overlap of p53 binding peaks enriched in TF-chRDP and ChIP-seq. **(C)** Venn diagram showing the overlap between the top 30% of TF-chRDP peaks and all ChIP-seq peaks. **(D)** Scatter plot showing peak density correlation between TF-chRDP and ChIP-seq in the 3,465 p53 ChIP-seq peak regions.

Following sequencing and data processing, we compared the TF-chRDP DNA loci with published ChIP-seq results (Sánchez et al., 2014) to validate the specificity of this novel method. The screenshots from the BASIC Browser demonstrate consistent DNA binding peaks at the p53 classical target gene regions, indicating high specificity (Figure 3A). Furthermore, a comparison of the p53 binding peaks with published data revealed that TF-chRDP identified more TF binding peaks, with over 95% of published peak regions included in TF-chRDP DNA peaks (Figure 3B). When the top 30% of TF-chRDP DNA peaks were selected and compared to ChIP-seq peaks, 75.3% of the reported loci overlapped (Figure 3C). Additionally, the scatter plot demonstrates a strong correlation between the RPM read counts of the two methods (Figure 3D), with a correlation coefficient of 0.88, further validating the effectiveness of the TF-chRDP method in capturing transcription factor binding events.

### 4.3 Assessing the efficiency of TF binding RNA using the TF-chRDP method

**FIGURE 4:**
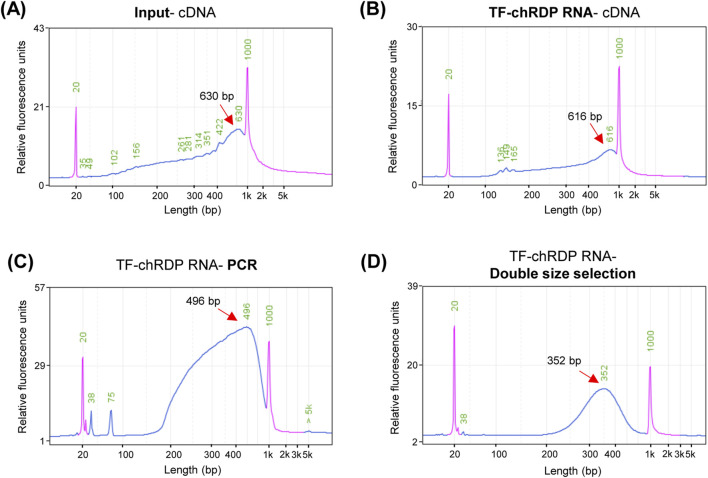
TF-chRDP RNA library preparation. **(A)** cDNA fragment size distribution after reverse transcription (RT) and second-strand synthesis of input RNA. **(B)** cDNA fragment size distribution after RT and second-strand synthesis of TF-chRDP RNA. **(C)** DNA fragment size distribution following PCR amplification of the TF-chRDP RNA. **(D)** DNA fragment size distribution after double size selection of the TF-chRDP RNA library. Red arrows indicate the peak positions, and the purple peak represents the marker.

The TF-chRDP method for RNA detection primarily involves extracting RNA from p53 antibody-enriched chromatin complexes, followed by reverse transcription using random hexamers to generate cDNA. In this study, RNA from chromatin complexes that were not subjected to antibody enrichment serves as a control. This cDNA undergoes second-strand synthesis, after which the profiling of double-stranded cDNA is assessed. The cDNA profiling obtained through the TF-chRDP method (Figure 4B) is consistent with that of cDNA from RNA that was not enriched (Figure 4A). These results demonstrate that the method is effective for both purifying TF-binding RNA and performing subsequent reverse transcription and cDNA synthesis.

The purified cDNA was then used as input for RNA library construction using Tn5 transposase, which simultaneously fragments the cDNA and adds sequencing adapters. Due to the low amount of p53-enriched RNA, we tested the optimal amount of Tn5 transposase for library construction with input cDNA, which exhibited a similar fragment size distribution. The Tn5 tagmentation, PCR amplification, and double size selection steps followed the same procedure as for the TF-chRDP DNA library, with the expected DNA size distribution between 250 and 600 bp (Figures 4C, D).

**FIGURE 5:**
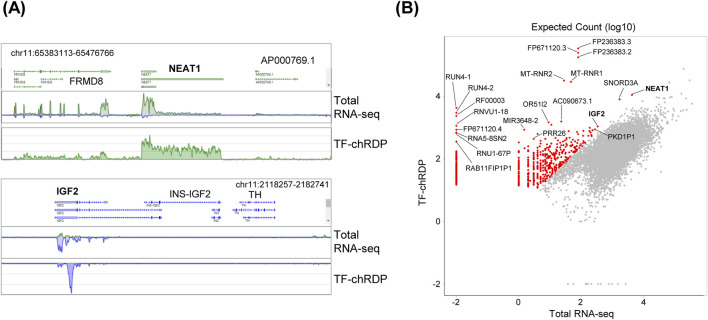
TF-chRDP RNA results show high specificity. **(A)** Screenshots of Basic Browser showing the comparison of TF-chRDP and ChIP-seq results at the p53 significantly enriched RNA regions, *NEAT1* (top) and *IGF2* (bottom). **(B)** Scatter plot showing the correlation of RNA expected count (log10) between TF-chRDP RNA and total RNA-seq, with significantly enriched RNAs (logFC >6) marked as red dots.

After data processing, we compared the specificity of the TF-chRDP RNA with total RNA-seq. We observed similar patterns between the two datasets, with notable enrichment in exonic regions (Figure 5A). The scatter plot shows the differential expression of genes between TF-chRDP RNA and total RNA, the significant enriched RNAs were used for further analyze (Figure 5B). These findings demonstrate the successful construction of a highly specific TF-chRDP RNA library with high efficiency.

### 4.4 Assessing the effectiveness of binding protein detection using the TF-chRDP method

Proteins were purified from p53 antibody-enriched chromatin complexes for mass spectrometry analysis, revealing a diverse array of proteins associated with p53. We identified a total of 298 p53-binding proteins using our novel method; however, only the top 10 most abundant proteins were presented in Table 1 to highlight high-confidence candidates that best demonstrate the effectiveness and specificity of our approach. The p53 protein ranked as the second most highly enriched protein, and several histone proteins from the chromatin complex were also enriched. Comparing these results with published data on p53-bound proteins, we found that proteins such as RUVBL2 (Wang et al., 2022), Lamin-A/C (Yoon et al., 2019), Ubiquitin (Brooks and Gu, 2011), and Heterogeneous nuclear ribonucleoprotein (HNRNP) (Shen et al., 2017) have all been reported to interact with p53. In addition to the top 10 proteins listed in Table 1, several other well-established p53-binding proteins were also detected, including HSP90 (Dahiya et al., 2019), Actin (Qi et al., 2020), Nucleolin (Daniely et al., 2002), HMGB1 (Štros et al., 2018), TRIM28 (Ding et al., 2023), Tubulin (Giannakakou et al., 2000), and SMARCA4 (Lee et al., 2002), among others. These findings highlight the specificity of protein identification using this novel TF-chRDP method.

**TABLE 1:** Top 10 p53-bound proteins identified by TF-chRDP method.

Protein IDs	Protein names	Gene names
P62805	Histone H4	HIST1H4A
P04637	Cellular tumor antigen p53	TP53
Q9Y230	RuvB-like 2	RUVBL2
P62807; Q99879; Q99877; Q93079; Q5QNW6; P58876; Q99880; O60814; P57053; Q96A08; A0A2R8Y619	Histone H2B type 1-C/E/F/G/I; Histone H2B type 1-M; Histone H2B type 1-N; Histone H2B type 1-H; Histone H2B type 2-F; Histone H2B type 1-D; Histone H2B type 1-L; Histone H2B type 1-K; Histone H2B type F-S	HIST1H2BC; HIST1H2BM; HIST1H2BN; HIST1H2BH; HIST2H2BF; HIST1H2BD; HIST1H2BL; HIST1H2BK; H2BFS
Q86YZ3	Hornerin	HRNR
P02545	Prelamin-A/C; Lamin-A/C	LMNA
Q99878; Q96KK5; Q9BTM1; Q16777; Q6FI13; P20671; P0C0S8	Histone H2A type 1-J; Histone H2A type 1-H; Histone H2A.J; Histone H2A type 2-C; Histone H2A type 2-A; Histone H2A type 1-D; Histone H2A type 1	HIST1H2AJ; HIST1H2AH; H2AFJ; HIST2H2AC; HIST2H2AA3; HIST1H2AD; HIST1H2AG
P62987; P62979; P0CG47; P0CG48	Ubiquitin-60S ribosomal protein L40; Ubiquitin; 60S ribosomal protein L40; Ubiquitin-40S ribosomal protein S27a; Ubiquitin; 40S ribosomal protein S27a; Polyubiquitin-B; Ubiquitin; Polyubiquitin-C; Ubiquitin	UBA52; RPS27A; UBB; UBC
P52272	Heterogeneous nuclear ribonucleoprotein M	HNRNPM
P25685	DnaJ homolog subfamily B member 1	DNAJB1

## 5 Discussion

This study presents the TF-chRDP method as a novel, high-efficiency technique for simultaneously capturing protein, RNA, and DNA bound to specific TFs. This method preserves the protein-DNA-RNA complex within the nucleus, which is crucial for maintaining chromatin structure and regulating gene expression, through a dual cross-linking process. TF-chRDP enables the simultaneous collection of protein, DNA and RNA in a single experiment. To minimize RNA degradation, we employed RNase-free consumables and thoroughly cleaned the workbench with RNase decontamination solution. Additionally, we used lightly decrosslinking conditions for RNA samples, in contrast to DNA samples, to prevent RNA damage. We used random hexamers as primers for reverse transcription, which allowed us to preserve RNA molecules from both coding genes and long non-coding RNAs (lncRNAs). Given that lncRNAs play key roles in protein binding and organizing chromatin architecture (Kaikkonen and Adelman, 2018), which is crucial for accurately profiling the protein-DNA-RNA complexes.

Due to the low amount of TF-chRDP DNA and RNA, we optimized the library preparation in several key ways. First, we utilized Tn5 transposase for both DNA and RNA samples, which simultaneously fragments the samples and adds sequencing adapters in a single step. Second, we tested the optimal amount of Tn5 transposase before formal tagmentation to ensure that most tagmented fragments fall within the 250–600 bp size range, improving the efficiency of double size selection for sequencing. Third, after Tn5 tagmentation, we quenched the reaction with a low concentration of SDS to release Tn5 without the need for extra DNA purification, making the process suitable for direct PCR amplification and minimizing sample loss. These optimizations improve efficiency, simplify the procedure, and save time in library preparation.

We selected the TF p53 as a test case and analyzed the bound protein, DNA and RNA. When comparing the TF-chRDP DNA to published ChIP-seq results, we observed more p53 binding peaks, which may be attributed to the dual-crosslinking procedure that preserved loci indirectly bound by p53. The results demonstrate a high degree of overlap between binding peaks, with a strong correlation in peak density. Furthermore, we evaluated the specificity of TF-chRDP RNA by comparing it to total RNA-seq. The results showed similar RNA profiling, with prominent enrichment in the exonic regions of genes. Furthermore, we analyzed the proteins identified in the p53-enriched protein-DNA-RNA complexes. The results included p53 and other previously reported proteins. These findings demonstrate the specificity and high quality of the protein, DNA, and RNA enriched using the novel TF-chRDP method.

This method can be adapted for use with other cells or tissues with minor modifications. In our lab, we have successfully applied this method to the GM12878 cell line and mouse brain tissue (data not shown), with similar effectiveness and specificity. However, there are key considerations when using this method. First, it is designed to analyze TF-associated chromatin complexes in the nucleus and is not suitable for capturing complexes primarily located in the cytoplasm. The protocol includes cell lysis, nuclear permeabilization, and washing steps before sonication, which removes most cytoplasmic contents. Only the nuclear fraction, which contains the TF-associated chromatin complexes, remains for the subsequent sonication and immunoprecipitation steps. Therefore, this method is not intended for capturing cytoplasmic complexes. Second, as mentioned, this method employs a 1% FA and 2 mM EGS dual crosslinking strategy, which enables the capture of all three biological molecules in a single trial. However, compared to milder crosslinking strategies, this approach may also incorporate some indirectly bound molecules. This novel method is particularly advantageous for studying multi-molecular interactions and chromatin structure functions, and high-confidence interactions can be identified through bioinformatics analysis. However, if the research goal is to focus on capturing a single molecular species, such as more precise and direct binding loci, traditional mild crosslinking methods and detection strategies may be more appropriate. Depending on the research goals, the crosslinking conditions (such as crosslinking time) can be further optimized.

This innovative method provides a robust platform for dissecting the intricate relationships between transcription factors, chromatin, RNA, and associated proteins. By elucidating these interactions, we can gain deeper insights into the regulatory networks that drive gene expression and cellular function, paving the way for potential therapeutic interventions targeting dysregulated transcriptional programs in diseases such as cancer.

## Data Availability

The original contributions presented in the study are included in the article/supplementary material, further inquiries can be directed to the corresponding authors.
